# Activating endogenous resolution pathways by soluble epoxide hydrolase inhibitors for the management of COVID‐19

**DOI:** 10.1002/ardp.202100367

**Published:** 2021-11-21

**Authors:** Manoj Manickam, Sangeetha Meenakshisundaram, Thanigaimalai Pillaiyar

**Affiliations:** ^1^ Department of Chemistry PSG Institute of Technology and Applied Research Coimbatore Tamil Nadu India; ^2^ Department of Chemistry Sri Krishna College of Engineering and Technology Coimbatore Tamil Nadu India; ^3^ Pharmaceutical Chemistry University of Tübingen Tübingen Germany

**Keywords:** anti‐inflammatory, COVID‐19, immune response, proresolving mediators, soluble epoxide hydrolase inhibitors

## Abstract

Anti‐inflammatory, specialized proresolving mediators such as resolvins, protectins, maresins, and lipoxins derived from polyunsaturated acids may play a potential role in lung diseases as they protect different organs in animal disease models. Polyunsaturated fatty acids are an important resource for epoxy fatty acids (EET, EEQ, and EDP) that mediate a broad array of anti‐inflammatory and proresolving mechanisms, such as mitigation of the cytokine storm. However, epoxy fatty acids are rapidly metabolized by soluble epoxide hydrolase (sEH). In animal studies, administration of sEH inhibitors (sEHIs) increases epoxy fatty acid levels, reduces lung inflammation, and improves lung function, making it a viable COVID‐19 treatment approach. Thus, using sEHIs to activate endogenous resolution pathways might be a novel method to minimize organ damage in severe cases and improve outcomes in COVID‐19 patients. This review focuses on the use of sEH inhibitors to activate endogenous resolution mechanisms for the treatment of COVID‐19.

## Graphical abstract

Polyunsaturated fatty acids are an important resource for epoxy fatty acids that mediate a broad array of anti‐inflammatory and proresolving mechanisms, such as mitigation of the cytokine storm. However, epoxy fatty acids are rapidly metabolized by soluble epoxide hydrolase (sEH). This review focuses on the use of sEH inhibitors to activate endogenous resolution mechanisms for the treatment of COVID‐19.

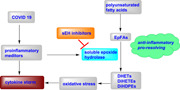

## 1 INTRODUCTION

The novel coronavirus (2019‐nCoV) first appeared in the Chinese city of Wuhan in December 2019, causing fever, dry cough, dyspnea, headache, pneumonia, and potentially progressive respiratory failure due to alveolar injury, as well as death.^[^ ^1^ ^]^ The World Health Organization renamed the current virus SARS‐CoV‐2 in February 2020 and coronavirus disease 2019 (COVID‐19) after sequence analysis revealed that it was closely linked to the SARS‐CoV strain from 2002 to 2003. Depending on pre‐existing diseases, severe COVID‐19 can have pathophysiological consequences for the heart, kidneys, liver, and central nervous system. Pre‐existing problems include arrhythmias, cardiac damage, liver dysfunction, renal failure, neurologic abnormalities, and encephalopathy.^[^ ^2^ ^]^

According to genome sequencing and phylogenetic research, severe acute respiratory syndrome coronavirus (SARS‐CoV), Middle East respiratory syndrome coronavirus (MERS‐CoV), and SARS‐CoV‐2 are all zoonotic illnesses that originated from bat coronaviruses and infected humans either directly or indirectly via an intermediate host. SARS‐CoV‐2 spreads faster than SARS‐CoV and MERS‐CoV, which are primarily transmitted by hospitals, with viral shedding occurring in asymptomatic patients before the development of symptoms. Asymptomatic transmission by patients increases the pandemic potential by several orders of magnitude.^[^ ^3^, ^4^ ^]^ As of May 15, 2021, the virus has infected over 162 million individuals and killed over 3.3 million people, according to the latest current data (https://covid19.who.int/). The extraordinary contributions of healthcare professionals, industrialists, and academic scientists have resulted in a wide range of vaccines, many of which have recently been approved and recommended for use in containing the global spread of SARS‐CoV‐2. The effectiveness of various vaccinations in reducing symptomatic illness is well known and proven (https://www.healthdata.org/covid/covid-19-vaccine-efficacy-summary). However, the vaccines' ability to deter infection remains a mystery.

Furthermore, the most recent strains that have emerged in several nations are only partially resistant (https://www.who.int/news-room/feature-stories/detail/the-effects-of-virus-variants-on-covid-19-vaccines). According to the US National Institutes of Health, remdesivir, an investigational medication originally intended as an RNA‐dependent RNA polymerase inhibitor against the Ebola virus, showed potential effectiveness in a clinical phase 3 study for COVID‐19. COVID‐19 patients who were hospitalized and administered remdesivir showed a 31% quicker recovery time (from 15 to 11 days). The WHO, however, does not recommend taking remdesivir regardless of the severity of the disease because there is no evidence that it improves lifespan or other problems in hospitalized patients (https://www.fda.gov/news-events/press-announcements/fda-approves-first-treatment-covid-19).

## 2 SARS‐COV‐2 INFECTION AND THE HOST IMMUNE RESPONSE

SARS‐CoV‐2 triggers the innate immune response as soon as the viral RNA is detected by endosomal receptors. As a result, Toll‐like receptors 7/8 and RIG‐I‐like receptors RIG‐I/MDA‐5 activate transcription factors, triggering a type I interferon (IFN) response. Infected cells are stimulated to control the early stages of virus invasion by producing proinflammatory cytokines and initiating an immune response as a result of the IFN‐I reaction. A cytokine storm (a strong immunological reaction in which the body releases too many cytokines into the bloodstream too rapidly) is a notable hallmark of acute respiratory distress syndrome (ARDS), which may induce multiorgan failure and death.^[^ ^5^ ^]^

Anti‐inflammatory and immunosuppressive therapies used alone or in combination may be promising to reduce pulmonary inflammation in acute respiratory conditions like ARDS. An example includes tocilizumab, a humanized immunoglobulin G1 (IgG1) anti‐IL‐6 receptor mAb used to treat rheumatoid arthritis and also licensed for the treatment of cytokine release syndrome. Tocilizumab has already completed multiple Phase 2 (NCT04317092 and NCT04315480) and Phase 3 (COVACTA and NCT04320615) trials, with promising preliminary results.^[^ ^6^ ^]^ Tocilizumab has also been approved in China for COVID‐19 critical patients who are suffering from severe respiratory failure.

The combination of bamlanivimab and etesevimab has been approved by the Food and Drugs Administration (FDA) for the treatment of moderate to severe COVID‐19 in adults and children. People older than 65 years of age as well as those with any chronic medical condition are eligible for treatment (https://www.fda.gov/news-events/press-announcements/coronavirus-covid-19-update-fda-authorizes-monoclonal-antibodies-treatment-covid-19-0). Bamlanivimab and etesevimab administered together have no adequate, approved or accessible alternatives for the affected population.

Infection prevention is complicated by the development of neutralizing antinucleoprotein IgG/IgM and anti‐RBD IgG/IgM antibodies. The transfer of antibodies from newly contaminated patients to new cases (plasma convalescent administration) was initially viewed as a solution for extreme COVID‐19 cases and received positive feedback. In March 2020, the FDA gave clinicians permission to consider and use convalescent plasma for the treatment of life‐threatening SARS‐CoV‐2 infections. However, data on seroconversion following SARS‐CoV‐2 infection, as well as its associated side effects, such as the risk of acute lung injury, are still limited.^[^ ^7^ ^]^

In chronically ill adults with COVID‐19, recent reports have called for the medicinal value of corticosteroids.^[^ ^8^ ^]^ A 10‐day regimen with low doses of dexamethasone (6 mg/day) decreased mortality in patients who required oxygen assistance, according to a report. However, the beneficial effect was not observed in patients who did not need oxygen. This finding further emphasizes the importance of tailoring therapy to the complex nature of COVID‐19 and using various techniques for the early stage of viral invasion versus the late stage, which is characterized by a strong inflammatory response.

Corticosteroids (e.g., prednisone or methylprednisolone) have been linked to both positive and negative clinical outcomes in patients with other pulmonary infections. In patients with *Pneumocystis jirovecii* pneumonia and hypoxia, prednisone treatment reduced the risk of death.^[^ ^9^ ^]^ In outbreaks of other novel coronavirus infections (e.g., MERS and SARS),^[^ ^10^ ^]^ corticosteroid therapy has been linked to virus clearance delays.^[^ ^11^ ^]^ The RECOVERY trial, a large, multicenter, randomized, open‐label trial (https://www.covid19treatmentguidelines.nih.gov/immunomodulators/corticosteroids/), is largely responsible for recommendations on the use of corticosteroids for COVID‐19.

The present review addresses activating endogenous resolution pathways by soluble epoxide hydrolase (sEH) inhibitors for the management of COVID‐19.

## 3 POLYUNSATURATED FATTY ACIDS (PUFA) AND THEIR ANTIVIRAL ACTIVITY

The stereoselective enzymatic conversion of PUFA such as arachidonic acid (AA), eicosapentaenoic acid (EPA), and docosahexaenoic acid (DHA) produces specialized proresolving mediators (SPMs) in the cells of the innate immune system.^[^ ^12^ ^]^ Under the inflammation condition, for example, AA is produced by membrane phospholipids through phospholipase‐A2.^[^ ^13^ ^]^ AA was reported to be effective against different microorganisms and enveloped viruses, including influenza and HIV.^[^ ^14^, ^15^, ^16^, ^17^, ^18^, ^19^, ^20^ ^]^ Staphylococci are killed by AA in the lungs' alveolar macrophages.^[^ ^21^, ^22^ ^]^ Das et al.^[^ ^23^ ^]^ suggest that AA acts as an endogenous antiviral compound to inactivate enveloped viruses, such as the influenza virus, HIV, or SARS‐CoV‐2. Recent research has found that SARS‐CoV‐2 disrupts AA metabolic pathways, perhaps resulting in an imbalance between proinflammatory AA metabolites like mid‐chain hydroxyeicosatetraenoic acids (HETEs) and terminal HETE (20‐HETE) and anti‐inflammatory metabolites like epoxyeicosatrienoic acids (EETs) and subterminal HETEs. On the basis of this interpretation, novel therapeutic strategies to modulate the level of endogenous anti‐inflammatory metabolites of AA and promote the patient's endogenous resolution mechanisms have been proposed to alleviate virus‐associated systemic inflammation and improve primary outcomes in COVID‐19 patients.^[^ ^24^ ^]^

The mechanism of the antimicrobial action of PUFAs may include their ability to induce leakage and even lysis of microbial cell membranes (including disruption of viral protein envelopes), as well as various cellular metabolic effects, including effects on transportation of amino acids, and uncoupling of oxidative phosphorylation.^[^ ^25^, ^26^, ^27^ ^]^ On the basis of this evidence, it was suggested that T and B cells, leukocytes, macrophages, and even other cells affected by viruses release PUFAs and can inactivate the invading microorganisms.^[^ ^23^ ^]^ This could be one of the fundamental mechanisms used by the human body to protect itself from various microbes. Hence, a deficiency in PUFA can make humans more susceptible to different viruses, including SARS‐CoV‐2, a causative agent in the ongoing COVID‐19 pandemic.^[^ ^23^ ^]^

Modifying key inflammatory pathways of the PUFA cascade can lead to the development of many COVID‐19 treatment methods.^[^ ^28^ ^]^ When human cells are exposed to SARS‐Co‐V‐2 and/or human coronavirus 229E, large quantities of PUFAs are released, inactivating the viruses. As a result, it is possible that the production of PUFAs by infected cells causes SARS‐CoV‐2 to multiply and induce COVID‐19.^[^ ^29^ ^]^

To combat SARS‐CoV‐2 and other illnesses, the usage of suitable quantities of PUFAs may result in the synthesis of pro‐ and anti‐inflammatory cytokines and bioactive lipids in an ordered and cohesive manner. A lack of PUFAs may impact an individual's development of COVID‐19. For example, a deficit of PUFAs is linked to “sepsis,” which is very similar to COVID‐19. COVID‐19 leads to higher mortality in people with diabetes, hypertension, and coronary heart disease, which can be ascribed to a shortage of PUFA.^[^ ^30^ ^]^

## 4 EICOSANOIDS AND SPECIALIZED PRORESOLVING MEDIATORS

Eicosanoids are PUFA‐derived lipid autacoids that include prostaglandins, thromboxanes, and leukotrienes, and are important mediators of inflammation, resolution, and tissue homeostasis.^[^ ^31^ ^]^ Eicosanoids are involved in a variety of physiological processes, including fever, allergy, and pain.^[^ ^31^, ^32^ ^]^ The proinflammatory effects of PUFA‐derived eicosanoids, prostaglandins, leukotrienes, and thromboxanes regulate a variety of homeostatic and inflammatory processes linked to a variety of diseases.^[^ ^33^ ^]^

**Figure 1:**
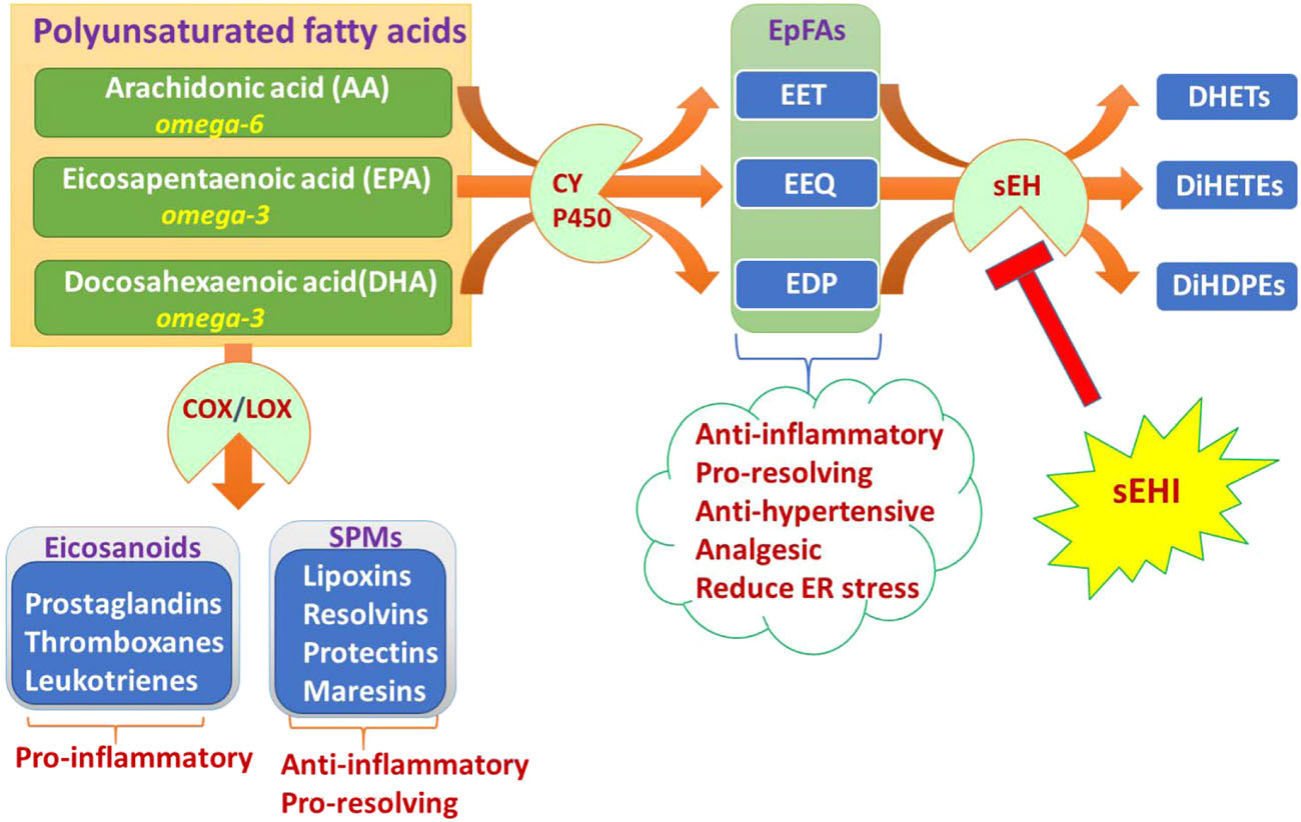
Polyunsaturated fatty acid pathway to derive eicosanoids, SPMs, and EpFAs. COX, cyclooxygenases; CYP450, cytochrome P450; DHET, dihydroxyeicosatrienoic acid; DiHDPE, dihydroxydocosapentaenoic acid; DiHETE, dihydroxyeicosatetraenoic acid; EpFA, epoxy fatty acid; EET, epoxyeicosatrienoic acid; EEQ, epoxyeicosatrienoic acid; EDP, epoxydocosapentaenoic acid; LOX, lipoxygenases; sEH, soluble epoxide hydrolase; sEHI, soluble epoxide hydrolase inhibitors; SPM, specialized proresolving mediators

SPMs are grouped into four families: lipoxins, resolvins, protectins, and maresins.^[^ ^12^, ^34^ ^]^ However, lipoxins, resolvins, protectins, and maresins have potent anti‐inflammatory actions. This anti‐inflammatory mechanism helps in wound healing and augments the phagocytic capacity of macrophages and other cells to remove debris from the site(s) of infection and injury and enhance microbial clearance (Figure 1).^[^ ^35^, ^36^, ^37^, ^38^, ^39^, ^40^, ^41^ ^]^

It is noteworthy that SARS‐CoV‐2, a causative agent of COVID‐19, may trigger a cell death‐induced eicosanoid storm, which in turn initiates a robust inflammatory response.^[^ ^42^ ^]^ In COVID‐19, uncontrolled cytokine levels, known as cytokine storms, have been identified as a major factor contributing to morbidity and mortality. However, the role of eicosanoids in COVID‐19 as key mediators of both inflammation and its active resolution by SPMs remains poorly explored.^[^ ^13^, ^23^, ^43^ ^]^ It is noteworthy that not all eicosanoids are proinflammatory, as PUFAs may be converted into anti‐inflammatory and proresolution docosanoids under the right circumstances.^[^ ^31^ ^]^ The cyclooxygenase (COX) inhibitors have received mixed reviews. Treatment with COX inhibitors was not linked to an increase in negative outcomes in COVID‐19 patients, according to a few investigations.^[^ ^44^ ^]^ However, a recent preprint research study recommends against using routinely prescribed nonsteroidal anti‐inflammatory medicines that are COX inhibitors in patients with coronavirus disease (COVID‐19).^[^ ^45^, ^46^ ^]^ To describe the specific risk of particular COX inhibitors in COVID‐19 patients, more studies are needed.

## 5 SPECIALIZED PRORESOLVING MEDIATORS AND COVID‐19

SPMs may play a new role in lung diseases because they actively stimulate the resolution of infectious inflammation and protect different organs in animal disease models.^[^ ^12^ ^]^ Despite regulating host responses, these endogenous mediators actively enhance the resolution of inflammatory response mechanisms such as reducing the production of proinflammatory cytokines and chemokines, limiting neutrophil trafficking, bacterial killing, and stimulating the macrophage phagocytosis of apoptotic cells and cellular debris via G protein‐coupled receptors.^[^ ^34^, ^47^, ^48^ ^]^ The antiviral activity of SPMs and their lipid precursors at nanogram doses in the setting of influenza without being immunosuppressive^[^ ^43^ ^]^ and the promotion of antiviral B‐cell antibodies and lymphocyte activity highlight their potential use in the fight against COVID‐19.^[^ ^49^ ^]^

## 6 EPOXY FATTY ACIDS (EpFAs) AND SPECIALIZED PRORESOLVING MEDIATORS

EpFAs may induce the production of SPMs by shifting PUFA metabolism to favor inflammation resolution.^[^ ^31^ ^]^ For example, EETs shift arachidonic acid metabolism to stimulate the production of SPM, such as lipoxins, which stimulate clearance of inflammatory cellular debris and counter proinflammatory cytokine production without being immunosuppressive.^[^ ^50^ ^]^

**Figure 2:**
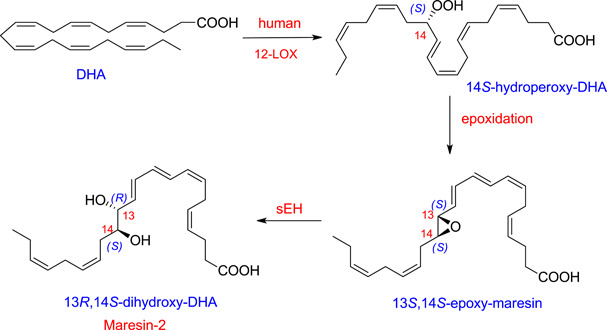
Biosynthesis of maresin‐2 from docosahexaenoic acid. DHA, docosahexaenoic acid; LOX, lipoxygenases; sEH, soluble epoxide hydrolase

On the contrary, maresin biosynthesis is initiated in macrophages by 14‐lipoxygenation of DHA by the action of human 12‐lipoxygenase (ALOX12 or ALOX15), producing the hydroperoxy intermediate 14*S*‐hydroperoxydocosa‐4*Z*,7*Z*,10*Z*,12*E*,16*Z*,19*Z*‐hexanoic acid that is subsequently converted into 13*S*,14*S*‐epoxy‐maresin. This is believed to undergo enzymatic hydrolysis to yield bioactive maresin‐1. In addition, 13*S*,14*S*‐epoxy‐maresin, which has important biological activities of its own, is the precursor for 13*R*,14*S*‐dihydroxy‐docosahexaenoic acid, designated maresin‐2, via the action of the sEH (Figure 2).^[^ ^51^ ^]^

13,14‐Epoxy‐maresin shows new mechanisms in the resolution of inflammation in its ability to inhibit proinflammatory mediator production by LTA4 hydrolase and to block arachidonate conversion by human 12‐LOX rather than merely terminating phagocyte involvement. 13,14‐Epoxy‐maresin also reduced arachidonic acid conversion by human 12‐LOX and promoted the conversion of M1 macrophages into the M2 phenotype, which produced more maresin‐1 from the epoxide than M1.^[^ ^51^ ^]^

## 7 sEH AND EpFA

EpFAs promote the active termination (resolution) of inflammation by mediating a broad array of anti‐inflammatory and proresolving mechanisms, including mitigation of the cytokine storm.^[^ ^43^, ^52^, ^53^ ^]^ However, the EpFAs are rapidly converted into dihydroxy derivatives by the enzyme sEH. Therefore, inhibition of sEH is a potential therapeutic approach for stabilizing EpFAs, which stimulates resolution of inflammation by promoting the production of proresolution mediators and preventing event cytokine suppression^[^ ^54^ ^]^ (Figure 1).

Although the major COVID‐19 clinical research focuses on antiviral and anti‐inflammatory strategies, the stimulation of inflammation resolution by sEHIs would be a novel host‐focused alternative to complement current therapies. For example, omega‐3 fatty acid (EPA and DHA)‐derived epoxyeicosanoids show anti‐inflammatory activity in various inflammatory diseases, including in the lung, heart, ocular angiogenesis, and pain.^[^ ^55^ ^]^ Omega‐3 epoxides (EEQ [epoxyeicosatrienoic acid] and EDP [epoxydocosapentaenoic acid]) stabilized by inhibition of sEH are important regulators of inflammation and autophagy in metabolic diseases.^[^ ^56^ ^]^ Thus, omega‐3 supplementation may also be synergized with sEH inhibition for suppressing inflammation.^[^ ^57^ ^]^

**Figure 3:**
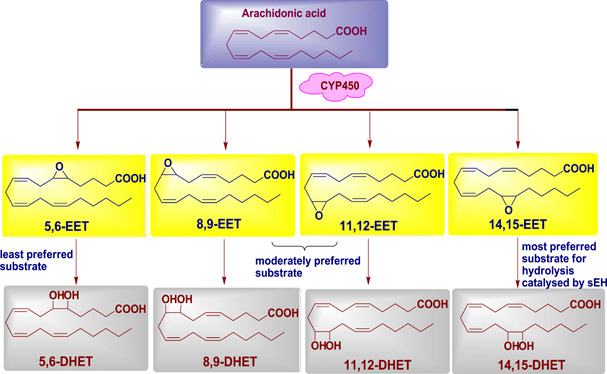
Role of soluble epoxide hydrolase enzyme in EETs. CYP450, cytochrome P450; DHET, dihydroxyeicosatrienoic acids; EET, epoxyeicosatrienoic acid; sEH, soluble epoxide hydrolase

Similarly, AA (an omega‐6 fatty acid) is a key starting molecule for all eicosanoids.^[^ ^58^ ^]^ Despite AA being a well‐known substrate for cyclooxygenases and lipoxygenases to yield eicosanoids, it is also a substrate for the cytochrome P450 pathway (Figure 2). One of the eicosanoid pathways converts AA into four regioisomeric EETs (5,6‐EET, 8,9‐EET, 11,12‐EET, and 14,15‐EET, Figure 3).^[^ ^59^, ^60^ ^]^ Among the EETs, 14,15‐EET is the most preferred substrate for hydrolysis catalyzed by sEH, while 5,6‐EET is the least preferred one.^[^ ^59^, ^60^ ^]^

## 8 sEHI AND RESOLUTION OF INFLAMMATION

sEHIs show potent anti‐inflammatory activity by inhibiting proinflammatory cytokines in various pathologic diseases, including hypertension, stroke, inflammatory bowel disease, atherosclerosis, diabetes, cerebral ischemia, immunologic disorders, ocular diseases, neurologic diseases, renal disease, organ damage, vascular remodeling, ischemia–reperfusion, lung disease (chronic obstructive pulmonary disease), fibrosis, graft stenosis, and other medical conditions.^[^ ^61^, ^62^, ^63^ ^]^

The inhibition of sEH has a plethora of beneficial central effects, such as reducing inflammation, endoplasmic reticulum (ER) stress, oxidative stress markers, tau pathology, and the amyloid burden.^[^ ^52^ ^]^ Besides peripheral inflammatory‐related diseases, inhibition of inflammation in the brain by targeting sEH was found to be a relevant therapeutic strategy for several psychiatric and neurological disorders.^[^ ^64^, ^65^ ^]^

According to growing data, sEH plays a critical role in the anti‐inflammatory characteristics of omega‐3 PUFA metabolism. The protein expression of sEH in the brains of mice with depressed‐like behavior, as well as in people with severe depressive illness, is greater than in controls, according to crucial data. When compared to controls, meta‐analysis studies in untreated depressed individuals have demonstrated an increase in the levels of inflammatory cytokines such as tumor necrosis factor (TNF‐α) and interleukin 6 (IL‐6). These findings are critical because cytokines can play a direct role in the development of depressive symptoms. Increased amounts of cytokines circulating in the periphery can, in the more permeable portions of the blood–brain barrier, affect the brain circuitry linked to depression symptoms. Cytokines, in particular, have been demonstrated to affect neurogenesis, a process that may be disturbed in depression and is essential for antidepressant effectiveness. On the basis of the findings, it was hypothesized that sEH inhibitor therapy had anti‐inflammatory, neurogenic, and antidepressant‐like effects in preclinical depression animals.^[^ ^66^ ^]^

Furthermore, inhibition of sEH has been used to treat rheumatoid arthritis by reducing nociception and inflammation,^[^ ^67^ ^]^ to reduce inflammation and fibrosis in the host myocardium by inhibiting sEH,^[^ ^68^ ^]^ and to improve vascular repair in the treatment of Kawasaki disease by inhibiting sEH.^[^ ^69^ ^]^ sEH inhibitors have recently been shown to be effective in the treatment of sepsis. Sepsis, also known as cytokine storm or inflammatory storm, is a life‐threatening condition caused by the reaction of the host's immune system to infection. A cytokine storm is an activation cascade that leads to autoamplifying cytokine production, which increases the severity of illnesses.^[^ ^70^ ^]^

As mentioned earlier, EpFAs induce the production of SPMs by shifting PUFA metabolism to favor inflammation resolution.^[^ ^31^ ^]^ As EpFAs are rapidly metabolized by sEH, administration of sEHIs can stabilize EpFA levels. This leads to the prevention of lung inflammation and improvement in lung function in animal models, making them a potential therapeutic strategy to fight against COVID‐19. Both SPMs and sEHIs downregulate the nuclear factor (NF)‐κB, the center of eicosanoid‐induced cytokine storms, which promotes proinflammatory cytokines and prostaglandin synthesis via cyclooxygenase.^[^ ^31^, ^71^, ^72^ ^]^

One of the key features of COVID‐19 infection is pathological thrombosis and clot removal. These can be attenuated by both SPMs and sEHIs, which promote the resolution of inflammation, thereby reducing ARDS, a life‐threatening complication associated with viral‐induced inflammation. Upon binding of the spike protein of SARS‐CoV‐1 or ‐2 to its receptor angiotensin‐converting enzyme 2 for host cellular entry, activation of ER stress transducer X‐box binding protein‐1 (XBP‐1) and upregulation of ER‐resident protein Herpud‐1 and chemokine CXCL2 have been observed in infected murine fibroblast L cells.^[^ ^73^ ^]^ Eventually, reduction of the ER stress response and enhancement of the activity of anti‐inflammatory, proresolving mediators may represent a promising therapeutic avenue in COVID‐19 treatment. For example, dexamethasone showed anti‐inflammatory activity in patients infected with COVID‐19.^[^ ^74^ ^]^ It was proposed that dexamethasone stimulates specialized proresolving mediators, which further promote the resolution of airway inflammation.^[^ ^71^ ^]^ Dexamethasone also reduces the ER stress response by promoting protein folding and degradation of misfolded proteins from the ER.^[^ ^75^ ^]^

**Figure 4:**
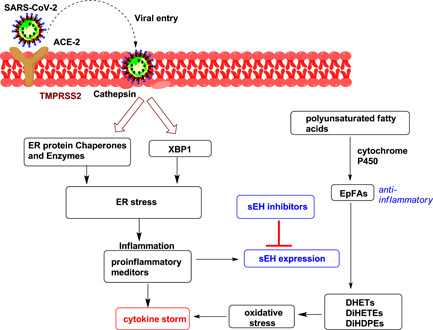
Multiple pathways to link cytokine storm and sEH expression. ACE‐2, angiotensin‐converting enzyme 2; DiHDPE, dihydroxydocosapentaenoic acid; DHET, dihydroxyeicosatrienoic acid; DiHETE, dihydroxyeicosatetraenoic acid; EpFA, epoxy fatty acid; ER, endoplasmic reticulum; sEH, soluble epoxide hydrolase; TMPRSS2, transmembrane protease serine‐2; XBP1, X‐box binding protein‐1

The ER stress response is suppressed in the heart and lungs by EpFAs derived from the cytochrome P450 family.^[^ ^76^, ^77^ ^]^ Although inflammation can induce sEH expression,^[^ ^78^ ^]^ COVID‐19 infection may further stimulate sEH levels throughout the body in various tissues. sEHIs stabilize the anti‐inflammatory, proresolving EpFAs, thus showing a potential therapeutic application in different preclinical studies and human trials, including sepsis, cancer, and cardiovascular and neuroinflammatory diseases.^[^ ^79^, ^80^, ^81^ ^]^ It is noteworthy that the administration of sEHIs suppresses pulmonary cytokine expression and neutrophil infiltration. This alleviates pulmonary inflammation and decreases mortality in murine models of endotoxin‐induced acute respiratory distress syndrome^[^ ^82^ ^]^ (Figure 4). Additionally, sEHIs reduce NF‐κB induction of inflammatory enzymes (i.e., COX‐2), as well as the downstream production of proinflammatory mediators, such as PGE2.^[^ ^71^ ^]^

The major cause of mortality associated with SARS‐CoV‐2 involves ARDS and the associated cytokine storm. The link between proinflammatory cytokine signaling and oxidative stress is being actively explored. For instance, it has been shown that overexpression of proinflammatory cytokines can induce an increase in oxidative stress.^[^ ^83^ ^]^ The cytokine storm occurs through the upregulation of cytokine expression via NF‐κB. After the establishment of this scenario, the cytokine storm induces oxidative stress via macrophage and neutrophil respiratory burst activity and the oxidative stress induces the cytokine storm.^[^ ^84^ ^]^

## 9 sEHIs IN CLINICAL TRIALS

**Figure 5:**
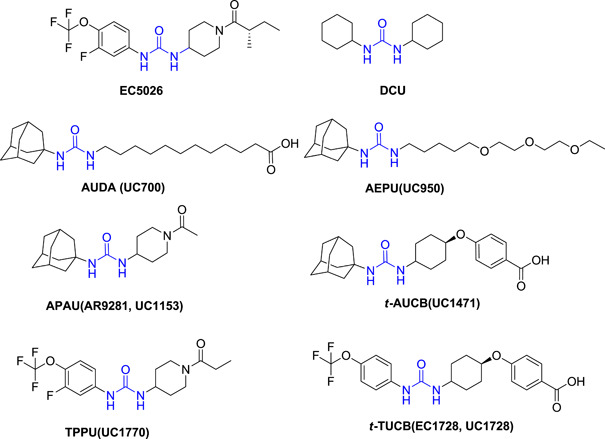
Representative ureas as potent soluble epoxide hydrolase inhibitors from early to clinical trial stages

A few potential sEHIs with urea functionality as a pharmacophore starting from early‐stage to clinical trials are shown in Figure 5. EC5026, a small‐molecule drug candidate under human phase 1a trials with no drug‐related events, is an exceptionally potent transition‐state mimic inhibitor of the sEH enzyme.^[^ ^85^ ^]^ EC5026 acts on the cytochrome P450 and stabilizes epoxides of PUFA, which are natural mediators that reduce pain, resolve inflammation, and maintain blood pressure. EC5026 mimics the slow tight‐binding transition state and inhibits the sEH at picomolar concentrations. EC5026 has been proven to be selective for the sEH enzyme in vitro when screened against a large receptor panel, suggesting that the potential for drug–drug interactions for this compound is low, further supporting its safety and its unique profile as a therapeutic for neuropathic pain. EicOsis Human Health, a pharmaceutical company, recently announced the successful completion of a Phase 1a single‐ascending dose clinical trial of EC5026, their oral drug candidate for the treatment of pain.^[^ ^86^ ^]^ In the early stages of sEHI discovery, Morisseau et al.^[^ ^87^ ^]^ found that the actual compound that acts as a potent, time‐dependent inhibitor against sEH is dicyclohexyl urea, a hydrated form of dicyclohexyl carbodiimide. The structure–activity relationship studies of the urea pharmacophore led to the development of numerous sEHIs. Among them, the adamantyl group used in N‐adamantanyl‐N'‐dodecanoic acid urea (AUDA) and 1‐adamantan‐3‐(5‐(2‐(2‐ethylethoxy)ethoxy)pentyl)urea yielded exceptionally high potency on the target sEH.^[^ ^88^ ^]^ AUDA could rescue the inhibition of proliferation and increased expression of Tumour Necrosis Factor alpha (TNF‐α), Interleukin‐1 beta (IL‐1β), Matrix Metalloproteinase‐9 (MMP‐9 induced by Kawasaki disease sera. Thus, AUDA could block the inflammatory response in Kawasaki disease.^[^ ^70^ ^]^ Treatment with AUDA reduced the protein expression levels of TNF‐α, matrix metalloproteinase, and IL‐1 in the Kawasaki disease mouse model, and the vascular repair by human coronary arterial endothelial cells (HCAECs) was markedly increased. These results suggest that AUDA positively modulates the vascular repair function of HCAECs in vitro and alleviates inflammation in heart tissue, demonstrating that AUDA could potentially be useful for the treatment of Kawasaki disease.^[^ ^70^ ^]^ The discovery of ureas with simple cyclic alkyl groups on one nitrogen atom and conformationally limited substituents on the other nitrogen atom of the urea scaffold was a major breakthrough. 1‐[(1‐Acetylpiperidin‐4‐yl)‐3‐adamantan‐1‐yl]urea (APAU) and trans‐4‐[4‐(3‐adamantan‐1‐yl‐ureido) cyclohexyloxy]benzoic acid (*t*‐AUCB) now have drug‐like characteristics, high potency, and better pharmacokinetics (PK) profiles as a result of this strategy.^[^ ^89^, ^90^ ^]^ APAU substantially reduced rotenone‐induced alterations in oxidative, proinflammatory, and apoptotic markers in in‐vitro research. APAU markedly reduced rotenone‐induced alterations in survival rate, negative geotaxis, oxidative stress, dopamine, and its metabolites in an in vivo investigation (*p* = 0.05). According to these findings, the chemical APAU provides considerable neuroprotection against rotenone‐induced Parkinsonism.^[^ ^91^ ^]^ Recently, it was discovered that *t*‐AUCB regulates ischemia arrhythmia by regulating microRNA (miR)‐1. The serum response factor, as well as the PI3K/Akt/GSK3 pathway, are both implicated in *t*‐negative AUCB's regulation of miR‐1, and both factors interact in this process. These findings offer a novel method and theoretical foundation for treating ischemia arrhythmia in the clinic.^[^ ^92^ ^]^

**Figure 6:**
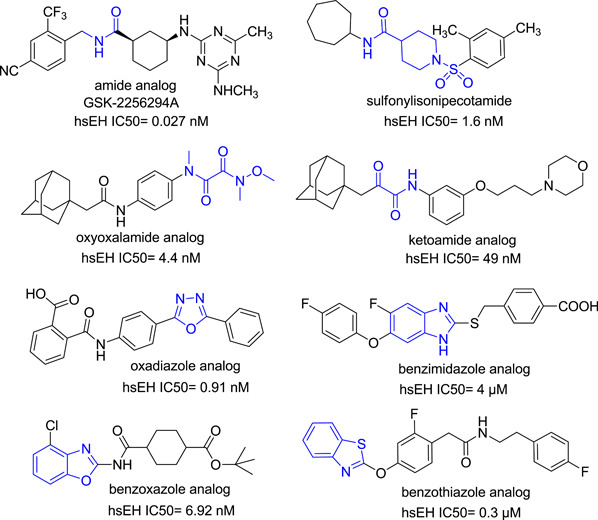
Representative non‐ureas as potent soluble epoxide hydrolase (sEH) inhibitors

1‐trifluoromethoxyphenyl‐3‐(1‐propionylpiperidin‐4‐yl) urea (TPPU) and trans‐4‐{4‐[3‐(4‐trifluoromethoxy‐phenyl)‐ureido]‐cyclohexyloxy‐benzoic acid (*t*‐TUCB) (UC1770, EC1728),^[^ ^90^, ^93^ ^]^ a metabolically unstable cyclic alkyl group, such as adamantyl, were replaced with a more stable one, *p*‐trifluoromethoxyphenyl. In diet‐induced obesity, *t*‐TUCB stimulates brown adipogenesis and lowers blood triglycerides. In vitro, *t*‐TUCB increased brown adipogenesis. Although *t*‐TCUB had no effect on body weight, fat pad weight, glucose, or insulin tolerance in obese mice, it did lower blood triglycerides and boost protein expression of lipid metabolism genes in brown adipose tissue.^[^ ^94^ ^]^ TPPU has established the industry standard for inhibiting sEH enzymes in several biological research investigations due to its good PK and high effectiveness on both primate and rodent sEH enzymes. A recent study found that peripheral pretreatment with TPPU reduced arthritis‐induced hypernociception and leukocyte migration in the temporomandibular joint (TMJ). Furthermore, TPPU decreased local proinflammatory cytokine concentrations while increasing the levels of the anti‐inflammatory cytokine interleukin‐10 in TMJ tissue.^[^ ^68^ ^]^ TPPU was found to decrease inflammation and fibrosis in another trial, making it a promising adjuvant to cardiac stem cell treatment.^[^ ^69^ ^]^ In a recent study, TPPU and EC5026 were found to inhibit neuroinflammation in the central nervous system of a lipopolysaccharide‐induced mouse model.^[^ ^95^ ^]^ Apart from the urea scaffold, many other functional groups have shown potent inhibition of the sEH enzyme. These are shown in Figure 6.

SK2256294A is an amide‐based, powerful, reversible, binding inhibitor of isolated recombinant human sEH (IC_50_ = 27 pM; *t*_1/2_ = 121 min), as well as the rat (IC_50_ = 61 pM) and mouse (IC_50_ = 189 pM) orthologues of sEH (IC_50_ = 189 pM). In a test utilizing a cell line transfected with the human sEH enzyme, GSK2256294A showed strong cellular inhibition (IC_50_ = 0.66 nM) of sEH.^[^ ^96^ ^]^ Clinical studies on GSK2256294A have recently been completed to determine how inhibition of sEH with GSK2256294 impacts tissue sEH activity and insulin sensitivity.^[^ ^97^ ^]^

Many additional scaffolds have been described as effective sEH inhibitors, including sulfonylisonipecotamide, oxyoxalamide, ketoamide, oxadiazole, benzimidazole, benzoxazole, and benzothiazole.^[^ ^96^ ^]^ The use of sEHIs is one area where their development has accelerated a wide variety of chemical scaffolds for multiple targets. The awareness that the balanced regulation of two targets can produce a greater therapeutic effect and side‐effect profile has sparked interest in the rational design of medicines that operate on particular multiple targets.^[^ ^98^ ^]^ The benzimidazole analog with ether and carboxylic units (Figure 6) was designed as an sEH/5‐LOX dual inhibitor that showed significant inhibition of carrageenan‐induced rat paw edema, a well‐defined model of acute inflammation.^[^ ^96^ ^]^ Similarly, the benzothiazole analog (Figure 6) was designed as a prototype dual inhibitor of sEH and leukotriene A4 (LTA4) hydrolase with submicromolar activity, providing promising options for future anti‐inflammatory agents.^[^ ^99^ ^]^

## 10 CONCLUSIONS

Resolvins, protectins, and maresins are SPMs that may play a new role in in the treatment of lung diseases because they promote anti‐inflammatory and proresolving mechanisms and protect different organs in animal disease models. PUFA are well‐known substrates for cyclooxygenases and lipoxygenases that yield proinflammatory eicosanoids, prostaglandins, thromboxanes, and leukotrienes, and anti‐inflammatory SPMs. On the contrary, PUFA are also metabolized by the cytochrome P450 to yield EpFAs that mediate a broad array of anti‐inflammatory and proresolving mechanisms, including mitigation of the cytokine storm. As EpFAs are rapidly metabolized by sEH, administration of sEH inhibitors can stabilize and increase EpFA levels, prevent lung inflammation, and improve lung function, making them a potential therapeutic strategy in the fight against COVID‐19.

## CONFLICT OF INTERESTS

The authors declare that there are no conflicts of interests.

## AUTHOR CONTRIBUTIONS

**Manoj Manickam, Sangeetha Meenakshisundaram, and Thanigaimalai Pillaiyar**: Final draft; writing—review & editing; visualization. **Manoj Manickam and Thanigaimalai Pillaiyar**: Conceptualization; writing—original draft; writing—review & editing; visualization.
